# IL-12-mediated transcriptional regulation of matrix metalloproteinases

**DOI:** 10.1042/BSR20171420

**Published:** 2018-06-12

**Authors:** Eugenia Roupakia, Georgios S Markopoulos, Evangelos Kolettas

**Affiliations:** 1 Laboratory of Biology, School of Medicine, Faculty of Health Sciences, University of Ioannina, Greece; 2 Biomedical Research Division, Institute of Molecular Biology and Biotechnology, Foundation for Research and Technology, Ioannina 45110, Greece

**Keywords:** Cytokines, IL-12, Matrix metalloproteinases, nuclear factor kappaB

## Abstract

Matrix metalloproteinases (MMPs) are extracellular matrix (ECM) remodelling enzymes involved in developmental processes, tissue remodelling and repair, inflammatory and immune diseases and cancer. In a recent issue of *Bioscience Reports* (vol. 37, issue 6, BSR20170973), Liu and colleagues investigated the expression of MMPs such as MMP-1 (interstitial collagenase), MMP-3 (stromelysin 1) and MMP-13 (collagenase 3) in human periodontal ligament fibroblasts (hPDLFs) regulated by interleukin-12 (IL-12), a cytokine implicated in inflammatory and immune responses. They showed that IL-12 activates canonical nuclear factor-κB (NF-κB) signalling leading to increased expression of MMP-1, MMP-3 and MMP-13, and to a smaller reduction in the expression of MMP-2 (gelatinase A) and MMP-9 (gelatinase B) at both mRNA and protein levels, with corresponding changes in the secreted levels of these ECM-remodelling and immune regulatory metalloproteinases. While canonical NF-κB signalling regulates these MMPs, it also interacts with additional factors to determine whether some of these MMPs are induced or downregulated, in response to IL-12. Here, we comment on the possible mechanisms of IL-12-mediated transcriptional regulation of MMPs.

Cytokines are the key regulators of inflammation and immunity, and modulation of their function may have enormous potential for therapeutic benefit in chronic inflammatory and autoimmune diseases. Type-I cytokines include the interleukin-6 (IL-6) and IL-12 families, which consist of structurally related four-helix bundle proteins. Unlike the members of the IL-6 family, which are secreted as single-subunit monomers, the IL-12 family members form heterodimeric complexes.

The IL-12 family is unique in comprising the only heterodimeric cytokines. IL-12 is a heterodimeric protein with a molecular weight of 70 kDa composed of two covalently linked subunits. Co-expression of the ligand-binding α subunit IL-12p35 (35 kDa) and the β subunit IL-12p40 (40 kDa), encoded by different genes localized on human chromosomes 3 and 5, leads to the formation of the biologically active p70 cytokine. The sequence of the p40 chain has a homology to the soluble extracellular domain of the membrane-bound receptors for IL-6 cytokines (IL-6 receptor; IL-6R) α-chain. This explains some of the redundant actions of these cytokines. IL-12 family subunits lack a transmembrane domain and are thus secreted as soluble α/β heterodimers. The IL-12 family consists of four cytokines with unique α/β subunit pairings: IL-12 (p35/p40), IL-23 (p19/p40), IL-27 (p28/Ebi3) and IL-35 (p35/Ebi3). Although structurally similar, IL-12 family members vary in function. Chain sharing, a characteristic of the IL-12 cytokine family, may also extend to the receptor usage with several cytokines utilizing the same receptor chains [1,2].

IL-12 (IL-12p70) is implicated in inflammatory and immune responses. IL-12 is secreted by antigen-presenting cells (APCs) such as macrophages, monocytes, dendritic cells (DCs), granulocytes and B cells in response to pathogenic microorganisms. IL-12 secretion is tightly regulated by several transcription factors. The *IL-12p35* gene is constitutively transcribed at low levels, but not translated. Following stimulation with microbial pathogen components, it is transcribed and its expression is amplified by NF-κB and interferon regulatory factors (IRFs) [2,3]. The *IL-12p40* gene promoter contains a number of transcription factor binding sites including NF-κB and Ets [4]. Microbial pathogen components are sensed by APCs such as DCs through toll-like receptors (TLRs). Importantly, selective production of each of IL-12 family member is regulated by triggering specific TLRs. TLR4 activation induces the production of both IL-12 and IL-23, whereas TLR2 activation induces IL-23 but not IL-12 [2,5].

Signalling through TLRs involves binding of TLRs to the adapter molecule MyD88 resulting in canonical NF-κB activation, which then induces the expression of genes encoding the subunits of IL-12 [2,6]. TLRs also activate the IRFs, IRF1, IRF3 and IRF7. Canonical NF-κB and IRF activation induce the transcription of IL-12p35 and IL-12p40. Subsequently, IL-12p70 is released and recognized by the IL-12 receptor (IL-12R) on natural killer (NK) and T cells [2].

The biological activities of IL-12 are mediated via binding to a membrane receptor complex (IL-12R) which is also composed of two subunits, IL12Rβ1 and IL12Rβ2, which are members of the class I cytokine receptor family, which includes IL-6, IL-11 and leucocyte inhibitory factor related to glycoprotein gp130 [2,7]. IL-12R is predominantly found on NK and T cells. IL12Rβ1 is required for high-affinity binding to the IL-12p40 subunit and it is associated with the Janus kinase (Jak) family member Tyk-2, while IL-12p35 binds to the IL12Rβ2 chain, associates with Jak-2 and mediates signal transduction via three tyrosine residues that act as a docking site for signal transducer and activator of transcription (STAT) 4 (STAT4), which is phosphorylated by JAK2. Thus, binding of IL-12 to the IL-12R complex, activates the JAK-STAT signalling pathway, with STAT4 being the predominant mediator of cellular responses activated by IL-12 [8–10]. Upon homodimerization and translocation to the nucleus STAT4 activates IFN-γ transcription. A positive feedback loop is established whereby IL-12-induced IFN-γ production by NK/T cells primes additional APCs for IL-12 production through IRF1 and IRF8 and IFN-γ-induced activation of T-bet, a T-box transcription factor expressed in CD4^+^ T cell promotes their differentiation to type 1 T helper (TH1) cells which express IL-12Rβ2 [2,3,11]. Moreover, IL-12-dependent binding of the transcription factor, activator protein-1 (AP-1) has also been shown [2].

Since IL-12 stimulates TH1 differentiation, it has been suggested that enhancing IL-12 activity in cancer may lead to an increased TH1 response and augmentation of the antitumour activity of the immune system. Activation of cytotoxic cells like NK cells and cytotoxic T lymphocytes (CTLs) is thought to result in increased extinction of tumour cells. IL-12 can also inhibit neo-angiogenesis and might therefore reduce the vascularization of growing tumours resulting in tumour cell necrosis [2].

IL-12 has also been implicated in the regulation of matrix metalloproteinases (MMPs) [10]. MMPs constitute a multigene family of zinc- and calcium-dependent ECM remodelling endopeptidases and chemokine regulators involved in several physiological and pathological processes. These include morphogenesis and developmental processes, tissue remodelling and repair, wound healing, inflammatory and autoimmune diseases such as periodontal diseases, arthritis, cardiovascular diseases and cancer [12–17].

Studies showed that synovial MMP-1 [18] and MMP-3 [19] levels correlated with IL-12 expression in canine rheumatoid arthritis. Expression levels of MMP-13 and IL-12 were also found to be correlated in experimental osteoarthritis [20].

Previous studies showed that IL-12 did not affect either the *MMP-2* or *MMP-9* mRNA or protein expression in the human monocytic U-937 cell line [21]. Injection of recombinant murine IL-18 or IL-12 alone or in combination significantly increased the levels of MMP-9 in mouse lung tissues, but no mechanistic details were provided [22]. However others, employing the human choriocarcinoma cell line, JEG-3, showed that IL-12 reduced the mRNA and protein expression levels, and also the enzymic activity of both MMP-2 and MMP-9, leading to suppression of tumour cell motility and invasion [23,24]. IL-12 treatment increased IFN-γ production in this setting [23]. In a murine model of breast cancer, IL-12 treatment reduced the levels of MMP-9, but not MMP-2, and it also reduced tumour cell production of VEGF by up-regulation of IFN-γ production leading to the suppression of tumour angiogenesis [10,25,26]. In an *in vivo* tumour model, it was shown that that the protein, mRNA expression and/or activity of MMP-2, MMP-3, MMP-7 and MMP-9 were significantly higher in UVB-exposed skin and tumours of IL-12 knockout mice compared with wild-type mice [27]. Collectively, these studies showed that IL-12-mediated production of IFN-γ led to the suppression of the expression of MMP-2 and MMP-9 in different cell types. However, whether NF-κB signalling was activated was not investigated. In line with this, it was also shown by employing IKK-null mouse embryo fibroblasts, that a subset of IFN-γ-responsive genes was dependent on the upstream activating NF-κB kinases, IKKα and IKKβ, but independent of NF-κB activation. In this setting, there was no defect in IFN-γ-stimulated STAT1α activation [28].

Studies have shown that cytokine-mediated regulation of MMP expression is complex involving the interplay of several transcription factors including mainly AP-1, NF-κB and STAT, and also activator protein-2 (AP-2), CREB and CCAAT/enhancer binding protein (C/EBP) [29–32] (Figure 1).

**Figure 1:**
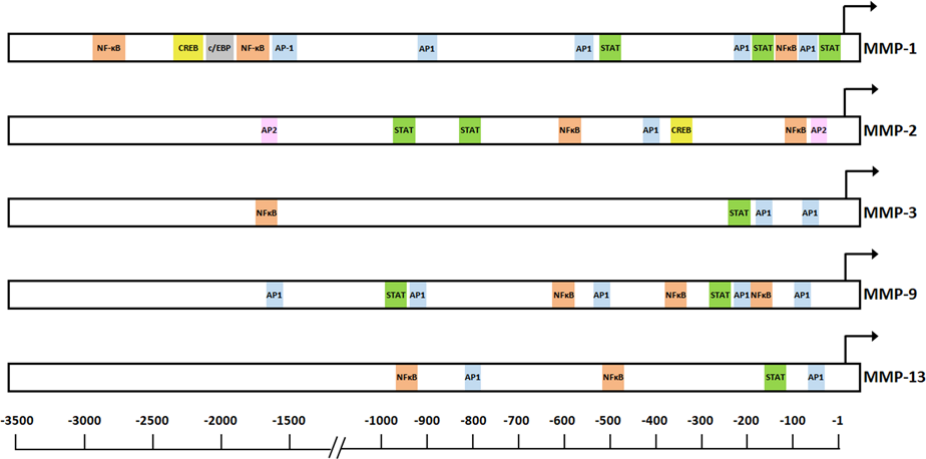
*cis*-Regulatory elements in the promoter regions of human *MMP-1, MMP-2, MMP-3, MMP-9* and *MMP-13* genes Transcription start sites are indicated with an angled arrow and the relevant *cis*-regulatory elements are represented within boxes. Data are compiled from the following references ([29,31,32] and references in the text).

The induction of the expression of several MMPs including MMP-1, MMP-3 and MMP-13 by pro-inflammatory cytokines in several cell types including fibroblasts [33,34–37] was shown to depend on extracellular signal-regulated kinase 1 and 2 (ERK1/2) and mitogen-activated kinase (MAPK) p38 mediated activation of canonical NF-κB, and to a lesser extent on the activation of other transcription factors such as AP-1, although these transcription factors co-operate to enhance MMP transcription [33,37–46].

In a different study employing articular chondrocytes, it was shown that MMP-1 and MMP-13 are differentially regulated by IL-1. IL-1 induction of MMP-13 required both AP-1 and NF-κB activation, while MMP-1 required only NF-κB activation [40,47–49].

Several studies have shown that the production of many inflammatory mediators and the production of MMPs depends on cytokine-mediated activation of MAP kinase leading to AP-1 transcription factor and of IKKβ-mediated canonical NF-κB activation [29–31,50,51]. For example, stimulation with LPS results in the activation of ERK1/2 and MAPK p38. Inhibition of MAPK p38 suppressed MMP-1 expression, but increased ERK activity and MMP-9 expression. This was because inhibition of MAPK p38 resulted in decreased binding of CREB and SP1 transcription factors to MMP-1 promoter, and to increased binding of NF-κB transcription factor to MMP-9 promoter. In contrast, inhibition of ERK1/2 suppressed MMP-1 and MMP-9 expression by inhibiting the binding of all AP-1, SP1 and NF-κB transcription factors to the promoters of both MMP-1 and MMP-9. Thus, LPS-induced production of MMP-1 is regulated by both ERK1/2 and p38, whereas MMP-9 production depends mainly on the ERK1/2-mediated activation of NF-κB [41,50].

In addition to the involvement of several transcription factors including SP1, AP-1 and NF-κB in the regulation of MMP gene expression, the MMP family members are also regulated by pro-inflammatory cytokines via STAT signalling [52,53]. For example, MMP-1 regulation involves binding of STAT3 to a proximal STAT-binding element (SBE) in the MMP-1 human gene promoter [54,55]. MMP-3 regulation by IL-6 involves a STAT3 binding to distal SBE element [56,57]. It was recently shown that IL-6 regulates MMP-1 expression, including MMP-1, MMP-2, MMP-3 and MMP-9, via proximal IFN-γ-activated site (GAS)-like SBEs involving binding of STAT1 and AP-1 but not STAT-3 [58–60]. Importantly, IFN-γ treatment resulted in the inhibition of MMP expression and also antagonized IL-6-dependent induction of MMP1 and MMP-3 gene expression, by reducing STAT1 binding to the respective MMP gene promoters [58]. IL-12 also leads to the activation of MAPK p38 and ERK1/2 and STAT phosphorylation [61–63].

Suppression of the expression of MMP-2 and MMP-9 by IL-12-mediated production of IFN-γ [10,23–27] may be due to STAT binding to GAS-SBEs in their respective gene promoters [61,62,64]. Previous studies showed that IFN-γ suppresses PMA-induced MMP-9 gene expression by activating the JAK-STAT pathway with p-STAT1α (Ser^727^) to bind to GAS, which is present in the promoters of IFN-γ-responsive genes. Genes that are negatively regulated by IFN-γ are some of the MMPs such as MMP-1, MMP-3, MMP-9 and MMP-13 [65–67]. Mechanistically, it was shown that IFN-γ-activated STAT1α suppresses MMP-9 gene transcription [68] by sequestration of the coactivators CBP/p300, without affecting binding of other transcription factors such as AP-1, SP1 and NF-κB to MMP-9 gene promoter [65,69]. Additional studies have shown a competition between IRF factors and NF-κB. For example, it was shown that IRF1 acts as competitive inhibitor of NF-κB binding to the MMP-9 promoter [70]. IL-12 was shown to induce the expression of IRF1 via STAT4 [71].

In summary, the regulation of MMP gene expression in response to pro-inflammatory cytokines involves the interaction of many transcription factors on MMP gene promoters. IL-12 can induce the activation of STATs leading to enhanced transcription of IFN-γ, which then suppresses MMP expression via GAS-SBEs such as in the case of MMP-2 and MMP-9, but it can also activate canonical NF-κB leading to the induction of MMP gene expression such as in the case of MMP-1, MMP-3 and MMP13. This differential expression of MMPs by IL-12-mediated IFN-γ production may be due to different STATs involved in MMP gene expression including STAT1, STAT3 and STAT4, and which of these co-operate with NF-κB to increase MMP gene expression or lead to sequestration of coactivators without affecting NF-κB binding to the MMP gene promoters. Alternatively, certain IL-12-induced IRF factors compete with NF-κB for binding to a MMP gene promoter.

## Abbreviations

APC: antigen-presenting cell
AP-1: activator protein-1
CREB: cAMP response element-binding protein
DC: dendritic cell
ECM: extracellular matrix
ERK1/2: extracellular signal-regulated kinase 1 and 2
Ets: E26 trasformation-specific
GAS: growth arrest specific
IKK: IκB kinase
IL-6: interleukin-6
IL-12: interleukin-12
IL-12R: IL-12 receptor
IFN-γ: interferon gamma
IRF: interferon regulatory factor
Jak: Janus kinase
LPS: lipopolysaccharide
MAPK: mitogen-activated protein kinase
MMP: matrix matalloproteinase
MyD88: myeloid differentiation primary response 88
NF-κB: nuclear factor-κB
NK: natural killer
SBE: STAT-binding element
Sp1: specificity protein 1
STAT: signal transducer and activator of transcription
TH1: type 1 T helper
TLR: toll-like receptor
